# Minimally invasive discectomy versus open laminectomy and discectomy for the treatment of cauda equina syndrome: A preliminary study and case series

**DOI:** 10.3389/fsurg.2022.1031919

**Published:** 2022-10-12

**Authors:** Morsi Khashan, Dror Ofir, Alon Grundshtein, Boris Kuzmenko, Khalil Salame, Dana Niry, Uri Hochberg, Zvi Lidar, Gilad J. Regev

**Affiliations:** ^1^ Department of Neurosurgery, Tel-Aviv Sourasky Medical Center, Tel-Aviv, Israel; ^2^ Sackler Faculty of Medicine, Tel Aviv University, Tel-Aviv, Israel

**Keywords:** cauda equina syndrome, open laminectomy, case series, minimally invasive, tubular discectomy

## Abstract

**Background:**

Cauda Equina syndrome (CES) is a potentially devastating condition and is treated usually with urgent open surgical decompression of the spinal canal. Currently, the role of minimally invasive discectomy (MID) as an alternative surgical technique for CES is unclear.

**Objective:**

The purpose of this study was to compare clinical outcomes following MID and open laminectomy and discectomy for the treatment of CES.

**Methods:**

The study cohort included patients that underwent surgery due to CES at our institute. Patients' outcomes included: surgical complications, length of hospitalization, postoperative lower extremity motor score (LEMS), Numerical Rating Scale (NRS) for leg and back pain, Oswestry disability index (ODI), and the EQ-5D health-related quality of life questionnaire.

**Results:**

Twelve patients underwent MID and 12 underwent open laminectomy and discectomy. Complications and revisions rates were comparable between the groups. Postoperative urine incontinence and saddle dysesthesia improved in 50% of patients in both groups. LEMS improved from 47.08 ± 5.4 to 49.27 ± 0.9 in the MID group and from 44.46 ± 5.9 to 49.0 ± 1.4 in the open group. Although, leg pain improved in both groups from 8.4 ± 2.4 to 3 ± 2.1 in the MID and from 8.44 ± 3.3 to 3.88 ± 3 in the open group, significant improvement in back pain was found only in the MID group. Final functional scores were similar between groups.

**Conclusions:**

Our preliminary results suggest that minimally invasive discectomy is an effective and safe procedure for the treatment of CES when compared to open laminectomy and discectomy. However, MID in these cases should only be considered by surgeons experienced in minimally invasive spine surgery. Further studies with bigger sample sizes and long-term follow-ups are needed.

## Introduction

Cauda Equina syndrome (CES) is a potentially devastating condition that can result in severe and permanent neurological deficits (1, 2). In the absence of trauma or an oncological condition, CES is most often caused by a giant disc herniation that occludes the spinal canal and severely compresses the thecal sac (3, 4). Severe possible consequences of this condition may include bowel and/or bladder dysfunction and motor weakness of the lower limbs. Therefore the recommended treatment option is urgent surgical decompression of the spinal canal, which includes the removal of the herniated disc fragment (5–7). Urgent surgical intervention has been found to be most effective in cases of incomplete neurological damage and when it is done within the first 48 h of presentation (3). Currently, the optimal surgical approach for the decompression of the spinal canal is still unclear. Several authors recommended the use of an open total laminectomy and discectomy in order to minimize chances of iatrogenic damage to the thecal sac and the neural elements while other authors reported that microdiscectomy neither increased the risk of postoperative complications nor resulted in incomplete decompression of the spinal canal (8, 9).

Minimally invasive discectomy (MID) was first described by Foley et al. (10) and has since gained acceptance as an alternative to traditional microdiscectomy. The limited trauma to the paraspinal muscles and posterior spinal ligaments has been shown to decrease post-operative back pain and thus enable faster mobilization and recovery (11–13).

It remains unclear whether outcomes of MID, for the treatment of CES, are comparable to those of open surgery. The aim of the present study was to compare Minimally invasive discectomy to open laminectomy and discectomy for the treatment of CES with regards to postoperative complication, recovery and overall quality of life.

## Methods

**Figure 1:**
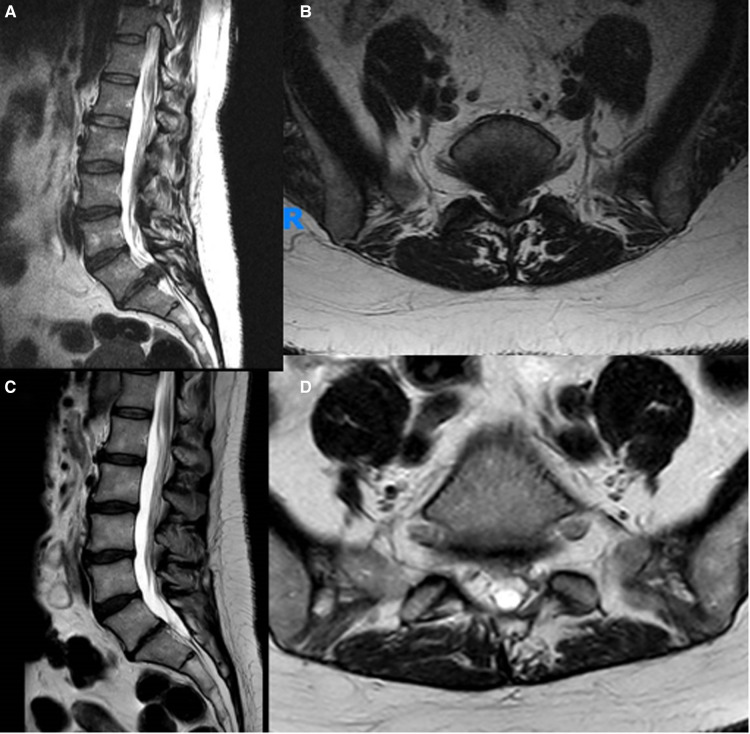
Preoperative (**A,B**) and postoperative (**C,D**) sagittal and axial MRI images of a typical patient diagnosed with an CES due to a giant L5-S1 disc herniation and operated by MID.

**Figure 2:**
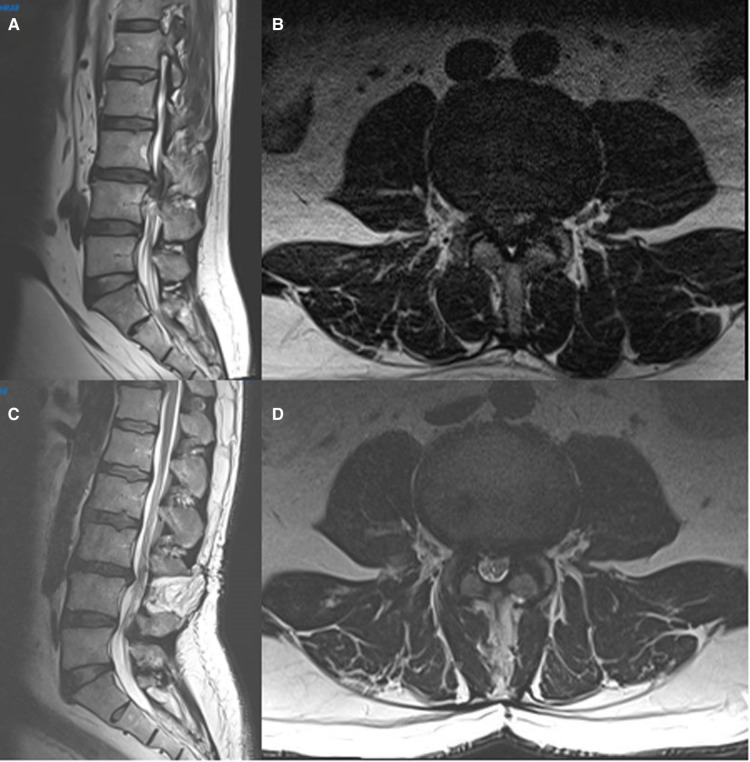
Preoperative (**A,B**) and postoperative (**C,D**) sagittal and axial MRI images of a typical patient diagnosed with an CES due to a giant L3–4 disc herniation and operated by open laminectomy and discectomy.

This is a retrospective analysis of prospectively collected data. The study was approved by our local Institutional Review Board and all patients provided informed consent before conducting the follow-up by phone interview. We collected medical records on all consecutive patients who underwent lumbar spine surgery due to CES between January 2010 and December 2019. Inclusion criteria included the diagnosis of CES due to lumbar disc herniation. The diagnosis of CES was determined by a combination of radiological evidence of a centrally herniated disc occluding the spinal canal and clinical symptoms that included: saddle anesthesia, low back pain, radicular pain, muscle weakness of the lower limbs, and acute bladder/bowel incontinence Figures 1, 2. The choice of surgical technique was made solely on the basis of the treating surgeon's preference and expertise.

Exclusion criteria included: spinal fracture, oncological pathology, or history of previous spinal surgery at the level of the current pathology.

Preoperative data included: demographic data, duration of clinical symptoms before surgery, and presenting symptoms. Radiological data included: spinal level of compression, and the type of disc pathology. Radiological analysis was conducted by a senior neuro-radiologist (D.N). Operative data included: operated spinal levels, and incidence of intraoperative complications. Measured clinical outcomes included: hospital length of stay (LOS), early postsurgical complications and revision surgery rates. Postoperative neurological outcomes included a subjective assessment of the patients improvement following surgery with regards to their urine inconstancy, dysesthesia and motor weakness. Additionally, the American Spinal Injury Association (ASIA) lower extremity motor score (LEMS) was used to evaluate objective lower-extremity motor function (14). This score grades motor function on a scale of 0 (no motor function) to 5 (full motor function) for each of the following 5 lower-extremity muscle groups. The LEMS has a maximum of 50 points (25 points per side). Pain and functional outcomes were assessed using the Numerical Rating Scale (NRS) for back and leg pain, Oswestry disability index (ODI) and health-related quality of life EQ-5D instrument.

### Surgical technique

**Figure 3:**
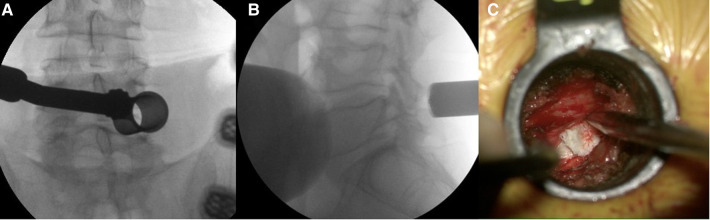
(**A, B**) Intraoperative fluoroscopy images showing the tubular retractor positioned at the L4–5 disc level. (**C**) Intraoperative view of the herniated disc underneath the retracted thecal sac and nerve root.

All the surgical procedures were performed in a single tertiary medical center by four senior spinal surgeons, experienced in minimally invasive spinal surgeries. MID procedures were done routinely under general anesthesia using an 18 or 20-millimeter tubular retractor system (METRx; Medtronic Sofamor Danek, Memphis, TN) and a surgical microscope. Surgery was performed through a unilateral approach. Using a diamond head high-speed drill, either an ipsilateral hemilaminotomy and medial facetectomy or a bilateral (“over the top”) decompression, was done. Once the lateral edge of the thecal sac was exposed, the smallest angled curettes (1.8 mm) and micro-pituitary rongeurs (2 mm) were used to extract and remove the disc fragment from underneath the thecal sac. Special attention was given to limit any retraction of the dura or nerve root in the initial part of the discectomy. Once a significant part of the disc fragment was removed, and the tension over the thecal sac lessened, a more liberal retraction was allowed in order to verify that all the disc fragments were completely removed and that the spinal canal was sufficiently decompressed (15) Figure 3.

Open procedures were routinely done with the use of magnifying loupes. Following exposure of the posterior elements of the spine, a total laminectomy and bilateral medial facetectomy of one or several levels was performed using an ultrasonic bone curette (BoneScalpel; Misonix Farmingdale, NY) and Kerrison rongeurs. In one case, a limited hemilaminectomy was performed in the open group. Following the full exposure of the thecal sac, removal of the herniated disc fragment was available from both sides of the spinal canal. A drain was placed in the surgical wound only in cases where the surgeon was concerned by the possibility of a post-operative hematoma.

### Statistical analysis

All data were analyzed using IBM SPSS Statistics for Windows, Version 23.0 (IBM, Armonk, NY, USA). Categorical variables were described as number. Continuous variables were described as mean and standard deviation. Categorical variables were compared between the two groups using Fisher's exact test and continuous variables were compared using Mann-Whitney test. Willcoxon test was used to compare pre- and post-surgical pain scores. All statistical testes were two-sided and *P* < 0.05 was considered as statistically significant.

## Results

### Patient characteristics and clinical presentation

The study cohort included a total of 24 patients, of whom 12 patients underwent MID and 12 patients underwent open decompression. Eighteen patients were males and six were females. The mean age was 44.2 ± 15.9 years in the MID group and 43.1 ± 11.2 years in the open surgery group (*P* = 0.19). No significant differences were found between the groups regarding previous spine surgeries, smoking or other systemic co-morbidities. The mean elapsed time from the initial presentation of symptoms until CES was diagnosed was not statistically different between the groups. 2.5 ± 3.1days in the MID group and 3.9 ± 3.9 days in the open surgery group (*P* = 0.45). No statistically differences were found when comparing the neurological presentation of patients in both groups. In the MID group 58% of patients presented with urinary incontinence, 66.7% with motor weakness and 66.7% with saddle anesthesia. In comparison, in the open surgery group 75% of patients presented with urinary incontinence (*P* = 0.67), 90.9% with motor weakness (*P* = 0.31) and 66.7% with saddle anesthesia (*P* > 0.99) Table 1.

**Table 1:** Demographic and clinical presentation.

	MID (*N*= 12)	Open (*N* = 12)	*P*-value
Gender (Male)	7 (53%)	11 (91%)	0.05
Age- mean (years)	44.2 (34.0–54.3)	43.1 (35.9–50.2)	0.19
Previous spine surgey	1 (8.3)%	3 (25%)	0.30
Smoking	1 (8.3)%	2 (17%)	0.50
Cerebrovascular	0 (0%)	1 (8.3)%	0.50
CRF	0 (0%)	0 (0%)	–
Neoplasia	1 (8.3)%	0 (0%)	0.50
Hypertension	3 (25%)	2 (17%)	0.50
Diabetes	1 (12.5%)	2 (17%)	0.50
Cardiovascular	0 (0%)	2	0.48
Pulmonary	0 (0%)	1 (10%)	0.50
Endocrine	0 (0%)	1 (10%)	0.50
Hematology	1 (8.3)%	0 (0%)	0.50
Depression/anxiety	1 (8.3)%	0 (0%)	0.50
**Clinical presentation**			
Length of complaints before CES diagnosis (days)	2.5 (0.5–4.5)	3.9 (1.4–6.4)	0.45
Back pain	10 (83.3%)	9 (75%)	0.5
Leg pain	10 (83.3%)	8 (67%)	0.37
Urinary incontinence	7 (58%)	9 (75%)	0.67
Bowel incontinence	1 (12.5%)	2 (17%)	0.5
Limb hypoesthesia	10 (83.3%)	10 (83.3%)	–
Saddle anesthesia	8 (66.7%)	8 (66.7%)	–
Motor weakness	8 (66.7%)	10 (90.9%)	0.32
Follow up time (months)	36.17 (14.9–57.5)	38.17 (17.9–58.4)	0.90

### Radiological analysis

Fifteen patients underwent an MRI study of the lumbar spine, the other 9 patients had a CT scan to confirm their diagnosis. For patients that presented with a clear clinical picture of CES an urgent lumbar spine CT scan was routinely done upon arrival to the Emergency Room (ER). If the CT findings were sufficient for the diagnosis the attending surgeon could elect to proceed immediately to surgery and avoid the delay until an additional MRI study will be done. Herniated discs were most commonly located at L5–S1 level (9 cases) followed by L4–5 and L3–4 (8 cases each). Disc herniations at the T12–L1, L1–2 and L3–4 were found in one patients each. Radiographic details of the intervertebral disc pathology are shown in Table 2. No statistically significant differences were found in type of disc pathology, the spinal levels of the herniation or the incidence of accompanied spondylolysis or spondylolisthesis.

**Table 2:** Preoperative radiological data.

	MID (*N* = 12)	Open (*N* = 12)	*P*-value
**Level of disc**			0.19
T12–L1	0 (0%)	1 (8.3%)	0.5
L1–2	0 (0%)	1 (8.3%)	0.5
L2–3	1 (8.3%)	1 (8.3%)	–
L3–4	3 (25%)	5 (42%)	0.67
L4–5	1 (8.3%)	6 (50%)	0.07
L5-S1	7 (58%)	2 (17%)	0.09
**Disc pathology**			
* *Bulge	0	0	–
* *Protrusion	1 (9.1%)	2 (18.2%)	5
* *Extrusion	8 (63.6%)	7 (63.6%)	0.5
* *Sequestration	3 (25%)	3 (25%)	–

### Surgery

Surgery was performed within 19.7 ± 16.6 h of the diagnosis in the MID group compared to 30 ± 13.9 h in the open group (*P* = 0.35). In two cases, a bilateral (“over the top”) decompression was done in the MID group through a unilateral approach due to concomitant lumbar stenosis at the same spinal level. In one patient in the MID group the minimally invasive approach was converted to an open laminectomy due to a large dural tear. However, the number of accidental dural tears was not significantly different between the groups Table 3. Mean LOS was 3.75 ± 2.8 days in the MID group compared to 6.1 ± 3.5 days in the open group, showing a strong trend towards shorter admissions for the MID group (*P* = 0.059). Two patients in the open group presented with recurring radicular symptoms following surgery due to recurrent and adjacent disc herniations. Both underwent revision surgeries with satisfactory results Table 3.

**Table 3:** Surgical data and complications.

	MID (*N* = 12)	Open (*N* = 12)	*P*-value
Time from presentation to surgery (hours)	19.7 (9.1–30.2)	30 (21.1–38.9)	0.75
Drains	1 (8.3%)	6 (54.5%)	0.07
Durotomy	1 (8.3%)	2 (17%)	0.50
Recurrent disc herniation	2 (17%)	1 (8.3%)	0.50
Revision surgeries	1 (8.3%)	1 (8.3%)	–
Conversion to open surgery	1 (8.3%)	–	
Medical complications	2 (17%) Pneumonia	1 (8.3%) Deep vein thrombosis	>0.99
Post-operative length of stay (days)	3.75 ± 3	6.1 ± 2	0.06

### Post-operative outcome

Mean follow up time was 36.17 ± 33.53 months for the open group and 38.17 ± 31.9 months for the MIS group (*P* = 0.9). Both groups reported similar improvement in their urinary incontinence, saddle dysesthesia, leg dysesthesia and motor deficits following surgery. At the final follow-up, five patients in the MID group reported no bladder dysfunction compared to six in the open group (*P* = 0.68) Table 4.

**Table 4:** Patient self-reported neurological outcomes.

	MID (*N* = 12)	Open (*N* = 12)	*P*-value
**Urine inconstancy**			
Pre-operative	7 (58%)	9 (75%)	
**Immediate post operatively**			
* No change*	8 (67%)	6 (50%)	0.29
* Partial improvement*	0 (0%)	1 (8.3%)	
* Complete improvement*	4 (33%)	5 (41.7%)	
3 months post operatively			
* No change*	6 (50%)	6 (50%)	0.44
* Partial improvement*	2 (18.2%)	0 (0%)	
* Complete Improvement*	4 (33%)	6 (50%)	
6 months post operatively			
* No change*	6 (50%)	6 (50%)	0.44
* Partial improvement*	2 (18.2%)	0 (0%)	
* Complete improvement*	4 (33%)	6 (50%)	
12 months post operatively			
* No change*	6 (50%)	6 (50%)	0.57
* Partial improvement*	1 (8.3%)	0 (0%)	
* Complete improvement*	5 (41.7%)	6 (50%)	
**Saddle dysesthsia**			
Pre-operative	8 (67%)	8 (67%)	
**Immediate post operatively**			
* No change*	8 (67%)	5 (41.7%)	0.29
* Partial improvement*	4 (33%)	7 (58%)	
* Complete improvement*	0 (0%)	0 (0%)	
**3 months post operatively**			
* No change*	6 (50%)	6 (50%)	0.81
* Partial improvement*	4 (33%)	3 (25%)	
* Complete improvement*	2 (18.2%)	3 (25%)	
**6 months post operative**ly			
* No change*	5 (41.7%)	6 (50%)	0.19
* Partial improvement*	5 (41.7%)	1 (8.3%)	
* Complete improvement*	2 (18.2%)	5 (41.7%)	
**12 months post operatively**			
* No change*	6 (50%)	6 (50%)	0.68
* Partial improvement*	4 (33%)	2 (18.2%)	
* Complete improvement*	3 (25%)	5 (41.7%)	
**Limb dysesthesia**			
Pre-operative	10 (83%)	10 (83%)	
**Immediate post operatively**			
* No change*	7 (58%)	5 (41.7%)	0.90
* Partial improvement*	4 (33%)	6 (50%)	
* Complete improvement*	1 (8.3%)	1 (8.3%)	
3 months post operatively			
* No change*	5 (41.7%)	5 (41.7%)	0.59
* Partial improvement*	0 (0%)	3 (25%)	
* Complete improvement*	7 (58%)	4 (33%)	
6 months post operatively			
* No change*	4 (33%)	6 (50%)	0.34
* Partial improvement*	0 (0%)	1 (8.3%)	
* Complete improvement*	8 (67%)	5 (41.7%)	
12 months post operatively			
* No change*	4 (33%)	6 (50%)	0.66
* Partial improvement*	0 (0%)	0 (0%)	
* Complete improvement*	8 (67%)	6 (50%)	
**Motor weakness**			
Pre-operative	8 (58%)	10 (83%)	0.37
**Immediate post operatively**			
* No change*	7 (58%)	7 (58%)	–
* Partial improvement*	3 (25%)	3 (25%)	
* Complete improvement*	2 (18.2%)	2 (18.2%)	
**3 months post operatively**			
* No change*	7 (58%)	5 (41.7%)	0.68
* Partial improvement*	3 (25%)	5 (41.7%)	
* Complete improvement*	2 (18.2%)	2 (18.2%)	
**6 months post operatively**			
* No change*	5 (41.7%)	5 (41.7%)	0.34
* Partial improvement*	4 (33%)	4 (33%)	
* Complete improvement*	3 (25%)	3 (18.2%)	
**12 months post operatively**			
* No change*	5 (41.7%)	3 (25%)	0.87
* Partial improvement*	3 (25%)	5 (41.7%)	
* Complete improvement*	4 (33%)	4 (33%)	

**Figure 4:**
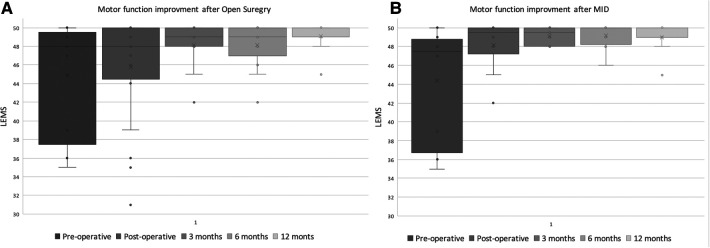
Distribution of reported lower extremity motor score (LEMS) of the MID (**A**) and open decompression (**B**) groups at preoperative, immediate postoperative, 3,6,12 months.

**Figure 5:**
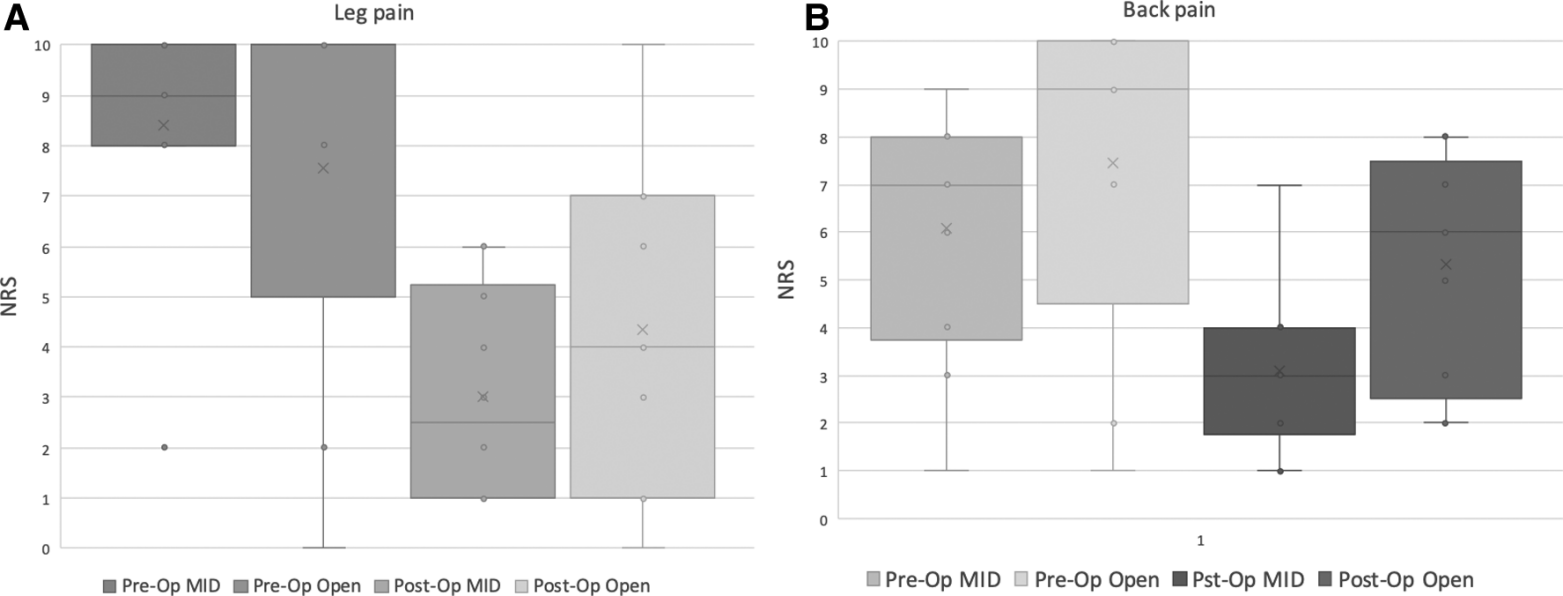
Distribution of reported rating scale (NRS) for preoperative and postoperative leg (**A**) and back (**B**) pain, in the MID and open decompression groups.

Similarly, LEMS scores improved in both groups following surgery. however, no significant difference between was found when the scores at presentation and at all the follow-up visits post-operatively were compared between the groups Figure 4. Postoperative leg pain showed significant improvement in both group. In contrast, significant improvement in back pain was found only in the MID group but not in the open group Figure 5. Functional outcome scores collected at the final follow-up did not show a statistically significant difference between the groups Table 5.

**Table 5:** Postoperative neurological and functional outcomes.

	MID (*N* = 12)	Open (*N* = 12)	*P*-value
**Follow up time (months)**	36.2 (14.9–57.5)	38.2 (17.9–58.4)	0.90
Baseline LEMS	47.1 (43.7–50.5)	44.5 (40.6–48.2)	0.11
Post op LEMS	48.2 (46.3–50.1)	45.7 (42.3–49.1)	0.10
3 m LEMS	44.7 (48.5–49.8)	49.8 (46.5–49.8)	0.96
6 m LEMS	49.2 (48.4–49.9)	47.9 (46.5–49.9)	0.06
12 m LEMS	49.3 (49.3–48.7)	49.0 (48.0–49.9)	0.75
Pre-operative leg pain NRS	8.4 (6.7–10.1)	8.4 (4.6–10.5)	0.58
post operative leg pain NRS	3.0 (1.5–4.5)	3.9 (1.7–6.9)	0.27
Pre-operative back pain NRS	6.1 (4.2–7.9)	7.4 (4.8–10.1)	0.40
Post operative back pain NRS	3.1 (1.8–4.4)	5.3 (3.4–7.2)	0.16
**Final follow up functional outcome**			
* *ODI	10.6 (1.0–20.1)	20.3 (8.9–40.8)	0.24
* *EQ-5D	6.0 (4.7–7.2)	8.2 (6.2–10.2)	0.15

## Discussion

CES is a relatively rare condition. Approximately 1%–2% of patients with lumbar disc herniation will develop a clinical presentation of acute cauda equina syndrome (16). As a result, the current scientific literature regarding the optimal medical treatment of this condition relies mainly on retrospective case series similar to the one presented herein.

Several studies focused on the clinical presentation of CES and on patients' outcomes following surgical intervention (17, 18). Special interest was given to the correlation between the timing of surgery and patients' post-operative neurological outcomes. Although most authors recommended urgent surgical decompression in this setting, critical analysis of the literature leading to this conclusion is not conclusive (5, 8).

Similarly, several authors claimed that optimal decompression should be achieved by a wide-open laminectomy followed by a discectomy (8). They argued this approach will decrease the risk of intra-operative complications including incidental dural tears and nerve root injury. Some authors also routinely supplement the laminectomy with an instrumented fusion in order to address post-operative iatrogenic instability or recurrent disc herniation (19). However, several studies reported CES could be successfully treated using a less invasive approach. Olivero et al. suggested that a unilateral hemilaminectomy and discectomy could produce similar results as total laminectomy (9). Successful decompression of large disc herniation causing CES was also reported using percutaneous endoscopic techniques (20, 21) and by using a minimally invasive tubular retractor system (22). However, all of these studies consisted of case reports or small retrospective case series without a control group.

Choosing an open laminectomy to treat CES has several inherent advantages over alternative surgical approaches that use a more limited approach. First, open decompression can usually be completed in a relatively short time which is especially important in these cases due to the emergent need to achieve adequate decompression of the thecal sac and in order to maximize the chances to reverse the neurological damage. Moreover, open laminectomy provides a superior exposure of the thecal sac with the option to remove extruded disc fragments from both sides of the spinal canal. Lastly, an initial wide decompression of the thecal sac could decrease the risk of nerve root injury and incidental durotomy during their retraction due to the initial decompression of the thecal sac achieved by the laminectomy. However, the potential disadvantages of open surgery include the relatively larger trauma to the paraspinal soft tissue, posterior ligamentous complex and facet joints. These injuries could be linked to an increased risk of post-operative complications including: surgical wound infection, epidural scarring and post-operative back pain (23, 24).

MID has have the potential of reducing these complication by minimizing damage to the paraspinal muscles and posterior bony spinal elements. In our experience, the risk of postoperative epidural hematoma formation even without the use of a drain is extremely low. It is however imperative to assure that the surgical wound is closed only after meticulous hemostasis has been achieved. MID is more technically challenging and usually requires a lengthy learning curve (25). In this study, Post-operative MRI studies in order to evaluate the efficiency of the decompression were not routinely order during the early post-operative period and thus were not available for this study. However, when comparing between MID and open laminectomy and discectomy we found that MID did not increase the risk of complications or of revision surgeries. Moreover, in one case the minimally invasive approach was aborted in favor of an open approach due to a large dural tear that could not be adequately addressed through the tubular retractor. The overall complication rates in both groups were similar to those previously published in the literature for open laminectomy and discectomy (23, 25). This low complication rate, especially in the MID group, may suggest that minimally invasive spinal decompression is an adequate technique to address CES. Moreover, back pain outcomes in the MID group were more favorable compared to the open surgery group as leg pain and functional outcomes showed a trend toward a greater improvement in the MID group that did not reach statistical significance. While the minimal surgical exposure could explain these more favorable pain and function outcomes, it should be recognized that these differences might be affected by confounding factors such as the small cohort size and selection bias of the two groups. It however demonstrates, at the very least, the non-inferiority of the MID group’s neurological outcome.

Due to the relative rarity of CES this current study has inherent limitations. The number of cases in our cohort albeit small is comparable to others in the literature (9, 22, 26). This factor in association with the usage of appropriate, but less sensitive, non-parametric statistical tests may lean towards a type 1 error. Moreover, our cohort was too small to identify specific risk factors for postoperative improvement. Additionally, evaluating sphincter dysfunction in this study was based on patients self-report without the use of a validated objective assessment tool. Despite these limitations, to date this is the first study that compares outcome of MID and open decompression for the treatment of CES. Additional prospective studies with larger cohorts are needed in order to validate these results.

In conclusion, while laminectomy may still be regarded as the safest surgical option for the treatment of CES, our findings show that MID is just as effective and might also provide superior results compared to open laminectomy and discectomy regarding back pain improvement.

## Data Availability

The raw data supporting the conclusions of this article will be made available by the authors, without undue reservation.
